# Differential expression of microRNA let-7b-5p regulates burn-induced hyperglycemia

**DOI:** 10.18632/oncotarget.20543

**Published:** 2017-08-24

**Authors:** Yajie Zhang, Bin Yin, Bin Shu, Zhen Liu, Hong Ding, Chiyu Jia

**Affiliations:** ^1^ Department of Burns and Plastic Surgery, The 309th Hospital of PLA, Beijing 100091, China; ^2^ Department of International Liaison, The 309th Hospital of PLA, Beijing 100091, China

**Keywords:** microRNA let-7b, miR-194, hyperglycemia, IGFIR, burn

## Abstract

One of the classical features observed in patients with burn injury is hyperglycemia. There have been previous reports that a cohort of microRNAs (miRNAs) is differentially expressed in the dermis of patients with burn injury. More specifically, it has been shown that the miR-194 can target the insulin-like growth factor receptor 1 (*IGF1R*) and silence its protein expression resulting in hyperglycemia. The objective of the current study was to discover if additional miRNA-mediated post-transcriptional mechanism exists that lead to suppression of IGF1R protein expression post-burn injury. Using the 30% total body surface area (TBSA) model of burn injury in rats we found that the miRNA let-7b can target *IGF1R* and downregulate its protein expression, in turn attenuating PI3K/Akt and Gsk3β activation leading to hyperglycemia. Increased let-7b expression was significantly more than the previously reported miR-194 both in the burn rats compared to sham and in patients with burn injury compared to healthy subjects. Serum from burn rats also resulted in decreased IGF1R protein expression in rat L6 myotubes. *In vivo* targeting of let-7b by antagomir mitigated the effect of increased let-7b expression on IGF1R protein expression and hyperglycemia. Thus targeting let-7b might be a promising approach to treat hyperglycemia in patients with burn injury.

## INTRODUCTION

Hyperglycemia is one of the classical features in patients with burn injury [1–3]. Mitochondrial dysfunction resulting from burn injury has been proposed to lead to skeletal muscle wasting and one of the leading cause of post-burn injury hyperglycemia since skeletal muscle is one of the hot-spot for glucose metabolism [4–5]. Indeed, it has been shown that administration of insulin can fasten wound healing in patients with burn injury by activating MAPK and phosphatidylinositol 3-kinase (PI3K)/Akt signaling pathway [6]. But the mechanistic details of post-burn hyperglycemia are not well defined.

Insulin is a main anabolic hormone involved in the regulation of glucose, protein and lipid metabolisms [7–9]. In some critical illnesses, intensive insulin therapy is utilized to combat the harmful effects of hyperglycemia. Most of the effects of insulin depend on its ability to activate the PI3K/Akt pathway, subsequently phosphorylate glycogen synthase kinase 3-β (GSK3-β), tuberous sclerosis protein-2 (TSC-2), bcl-2 antagonist of cell death (BAD), Forkhead box O (FOXO), etc. [7–9]. These Akt targets are important for the regulation of protein metabolism, inhibition of lipid lysis, promotion of fatty acid synthesis and glycogen synthesis. [7].

It has been recently shown that microRNAs (miRNAs), a type of endogenous, small noncoding RNAs, can functionally regulate diverse cellular and pathogenic processes. Through specifically binding and cleaving mRNAs or inhibiting their translation [10], miRNAs can function both as tumor suppressors or oncogenes and in a wide variety of diseases [11–13]. However, our understanding of the expression and function of miRNAs in burn induced hyperglycemia is still limited and deciphering the role of the same would be of significant clinical benefit. It has been recently shown that 66 miRNAs are differentially expressed post burn injury [14]. Furthermore, it has been established that miR-194 can target insulin-like growth factor 1 receptor (*IGF1R*) and result in hyperglycemia post-burn injury [15].

The objective of the current study was to evaluate if there are other post-transcriptional mechanism(s) mediated by miRNAs that regulate *IGF1R* expression post-burn injury which in turn causes hyperglycemia in patients with burn injury. Our results show that the miRNA let-7b is robustly induced in burn injury and attenuates IGF1R protein expression and downstream activation of GSK3β that results in hyperglycemia.

## RESULTS

It has been earlier shown that miRNA-194 can target *IGF1R* and silence its expression in burn cases. To identify if additional miRNAs are involved in targeting *IGF1R* expression by post-transcriptional regulatory mechanisms, we used two independent algorithms TargetScan [16] and microCosm [17] to *in silico* predict the miRNAs targeting the 3′ untranslated region (UTR) of human *IGF1R*. MiRNA-194 had 7 putative binding sites between nucleotides 559-565, 1021-1027, 1608-1615, 2370-2376, 4191-4197, 4599-4605, and 6813-6819. However, the preferential conserved targeting (P_CT_) values for all the seven sites (<0.1, NA, 0.12, NA, NA, NA, and <0.1; NA-cannot be calculated) were much less than 0.75. P_CT_ scores reflected the Bayesian estimate of the probability that a site is conserved due to selective maintenance of miRNA targeting rather than by chance or any other reason not pertinent to miRNA targeting [18]. And a P_CT_ <0.75 is usually considered an irrelevant miRNA-target prediction. Instead let-7b had three binding sites in the 3′UTR of *IGF1R* - 99-105 (P_CT_ = 0.74), 2619-2626 (P_CT_ = 0.93), 6661-6667 (P_CT_ = 0.89) sites with higher probability of preferential conservation (Supplementary Figure 1). Two of these three sites had P_CT_ > 0.75 and one just missed the cut-off with a P_CT_ of 0.74. MiR-15, miR-133a, and miR-30 also had P_CT_ > 0.75.

**Figure 1:**
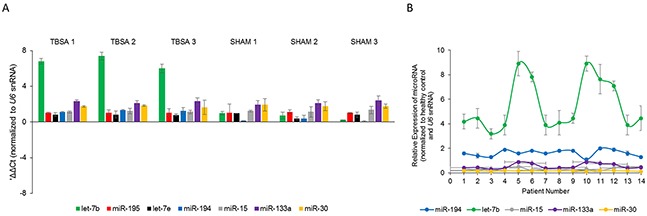
Expression of let-7b is induced post-burn injury **(A)** Steady state expression of indicated miRNAs in burn rats (TBSA) and sham rats (SHAM). Data is represented as mean ± standard deviation post-normalization to *U6* snRNA expression. **(B)** Fold changes in expression of indicated miRNAs in fourteen burn patients compared to healthy controls. Data is represented as mean ± standard deviation post-normalization to *U6* snRNA expression.

Using a 30% TBSA rat model, we first confirmed that fasting blood glucose levels were significantly higher in burn rats compared to sham rats (data not shown). Since it has been previously indicated that glucose metabolism by skeletal muscle is repressed post-burn injury [19], we used the anterior tibial muscle of the rat model at day 7 for determining miRNA expression levels. We assessed the levels of let-7b, let-7e, miR-194, miR-15, miR-133a, miR-15, and miR-195 (as a non-specific control) by real-time PCR. Whereas, miR-194 levels were increased 1.24 ± 0.1 folds in the burn rats, let-7b levels were increased 6.72 ± 0.7 folds in the burn rats, compared to sham rats (Figure 1A) (P<0.05). MiR-195, let-7e, miR-15, miR-133a, and miR-30 did not show any significant difference between burn rats and sham rats (Figure 1A) (P>0.05). This indicated that let-7b is perhaps a more potent mediator of burn-induced hyperglycemia in skeletal muscle compared to miR-194. Hence, we focused on let-7b in further experiments.

To confirm the potential role of let-7b in burn-induced hyperglycemia, we next evaluated let-7b, miR-194, miR-15, miR-30, and miR-133a expression in serum of 14 burn patients before surgical intervention and compared them to serum from 14 healthy subjects. Real-time PCR analysis showed that let-7b was significantly up-regulated in burn patients (5.49 ± 2.06 folds) compared with healthy subjects (Figure 1B; p = 0.018) (P<0.05). MiR-194 expression was also higher in serum from burn patients (1.62 ± 0.27) (Figure 1B; p = 0.039) (P<0.05), but the changes were much more with let-7b than miR-194. There were no significant changes in the expression of miR-15, miR-30, and miR-133a between healthy subjects and patients with burn injury. Cumulatively, our results indicated that let-7b expression is upregulated in burn patients, comparatively more than miR-194.

**Figure 2:**
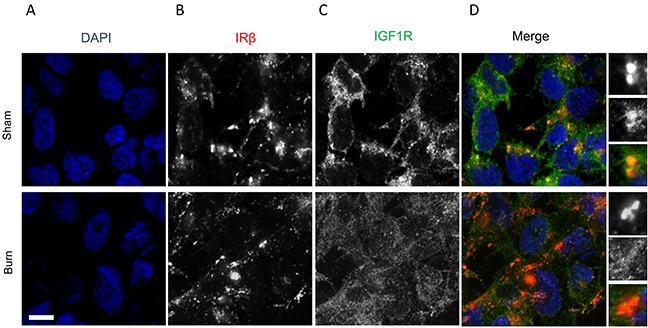
Immunofluorescence assay to detect relative expression of IRβ and IGF1R protein in anterior tibial muscle obtained from burn and sham rats at day 7 **(A)** DAPI staining, **(B)** IRβ, **(C)** IGF1R, and **(D)** merge images. Scale bar: 100 μm. Images are representative of at least 10 different images obtained within the experimental set.

Insulin, the key hormone involved in glucose metabolism, signals by binding through either IRβ or IGF1R. Immunofluorescence showed that IRβ expression was comparable in burn and sham rats (Figures 2A, 2B). But the IGF1R protein expression was significantly lower in burn rats compared to the sham rats (Figures 2C, 2D).

**Figure 3:**
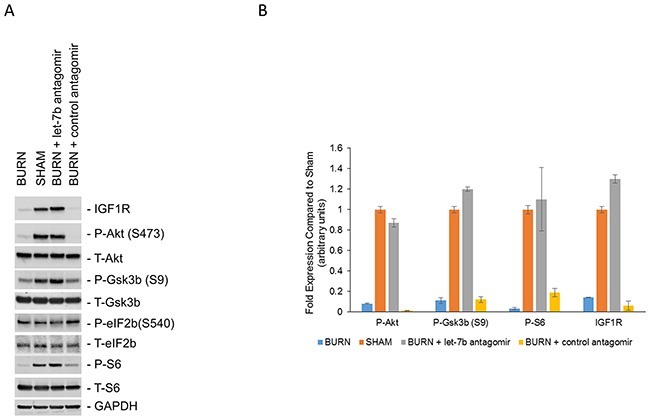
Impaired PI3K/Akt signaling in burn rats induced by high expression of let-7b **(A)** Western blot analysis of indicated proteins in lysates obtained from anterior tibial muscle in sham rats, burn rats, or burn rats pre-injected with anti-let7b antagomir or a control antagomir. The blots were stripped and re-probed with GAPDH as a loading control. Experiment is representative of four independent experiments. **(B)** Densitometric analysis of relative expression of P-Akt, P-Gsk3b (S9), P-S6, and IGF1R in the indicated conditions. Data is represented as means ± SD.

Since insulin signaling through IRβ or IGF1R activate PI3K/Akt signaling pathway, we determined levels of phosphorylated Akt, total Akt, phosphorylated Gsk3β, total Gsk3β, phosphorylated eIF2B, total eIF2B, phosphorylated S6, and total S6 in anterior tibial skeletal muscle of sham and burn rats (Figure 3, *left two lanes*). There were no obvious changes in phosphorylated eIF4B at serine 540 between sham and burn rats. However, IGF1R, phosphorylated Akt at serine 473, phosphorylated GSK3β at serine 9, and phosphorylated S6 kinase were significantly suppressed in the burn rats compared to the sham rats, indicating attenuation of PI3K/Akt and mTOR signaling pathway in the burn rats (Figure 3A, 3B) (P<0.05).

To confirm that the inhibition of PI3K/Akt-mTOR pathway is being mediated by increased let-7b levels targeting IGF1R, control or let-7b antagomir were injected through the tail vein of rat each day for 7 days before inducing burn injury. Three days post-burn injury, PI3K/Akt pathway activation was evaluated (Figure 3A, *right two lanes*). Suppressing let-7b expression by let-7b antagomir prevented decrease of IGF1R protein expression post-burn injury (Figure 3A, *lane 3*). Importantly, let-7b antagomir restored levels of phosphorylated Akt at serine 473, phosphorylated Gsk3β at serine 9, and phosphorylated S6 kinase to levels comparable to the sham rats (Figure 3A, *lanes 2 and 3*). Cumulatively, this indicated that let-7b directly targets *IGFIR in vivo* and attenuates PI3K/Akt pathway resulting in hyperglycemia.

**Figure 4:**
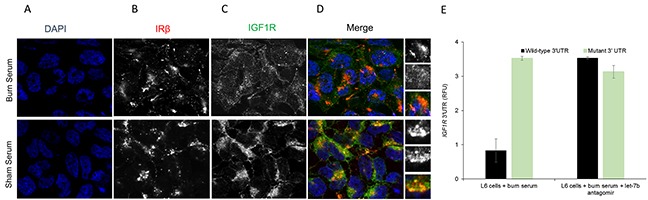
Immunofluorescence assay to detect relative expression of IRβ and IGF1R protein in L6 cells treated for 24 hours with serum obtained from sham rats and burn rats at day 7 **(A)** DAPI staining, **(B)** IRβ, **(C)** IGF1R, and **(D)** merge images. Scale bar: 100 μm. Images are representative of at least 10 different images obtained within the experimental set. **(E)** Relative luciferase activity of transiently transfected luciferase reporter constructs containing either full-length or mutated (let-7b binding site deleted) *IGF1R* 3′ UTR, alone or in combination with let-7b antagomir, in L6 myotubes treated with serum from burn rats. Data represents means ± SD of 3 independent experiments.

Finally, we wanted to evaluate if serum from burn rats was capable to transfer the effect of let-7b expression on IGF1R protein expression in L6 cells. L6 cells were treated with serum obtained from burn and sham rats at day 7 for 24 hours before IRβ and IGF1R protein expression was evaluated by immunofluorescence. As shown in Figures 4A, 4B exposure to either serum from burn or sham rats did not cause a change in IRβ expression. However, IGF1R expression was robustly downregulated in L6 cells exposed to serum from burn rats as compared to serum from sham rats (Figure 4C, 4D). Use of burn serum also attenuated PI3K/Akt activation as assessed by phosphorylated Akt at serine 473 and phosphorylated Gsk3β at serine 9 (*data not shown*). Cumulatively, this indicated that burn serum exposure can promote let-7b expression, which can attenuate IGF1R protein expression and PI3K/Akt signal pathway resulting in hyperglycemia.

To confirm that *IGF1R* is a bona-fide target within our experimental system, L6 myotubes treated with burn serum were transfected with renilla luciferase constructs harboring either wild-type or let-7b binding mutant (let-7b predicted sites deleted) *IGF1R* 3′UTR. Expression of renilla luciferase harboring wild-type, but not the mutant, *IGF1R* 3′UTR was significantly attenuated in L6 myotubes treated with serum from burn rats (Figure 4B) (P<0.05). However, on co-transfection of let-7b antagomir along with the luciferase reporter constructs expression from renilla reporters harboring either the wild-type or mutant *IGF1R* 3′UTR were comparable (Figure 4B). This proved that *IGF1R* is a bona-fide target of let-7b in the context of burn injury.

## DISCUSSION

Our results showed that the miRNA let-7b is robustly induced in both the 30% TBSA rat model of burn injury as well as in patients with burn injury. This induction was overwhelmingly more when compared to increase in expression of the previously reported miR-194 [15]. Furthermore, the high expression of let-7b attenuated PI3K/Akt activation and Gsk3β activation, which was proved by using anti-let7b antagomir *in vivo* which de-repressed the aforementioned signaling pathways in the sham rats.

Insulin is the critical hormone regulate glucose metabolism. Insulin can bid to both IRβ and IGFIR and activate downstream signaling pathway leading to activation of PI3K/Akt that in turn is the central regulator of glucose homeostasis [20]. Phosphorylated Akt and Gsk3β was thus evaluated as a measure of PI3K/Akt signaling activation. Phosphorylated-mediated activation of glycogen synthase kinase 3 beta (Gsk3β) functions as a negative regulator of glycogen synthesis and its inactivation leads to activation of glycogen synthase and increase glycogen synthesis [21]. Our results indicate that increased let-7b suppressing IGF1R activation ultimately leads to inactivation of Gsk3β and concurrent activation of glycogen synthase leading to hyperglycemia.

Our results also indicate that topical insulin application in patients with burn injury might alleviate hyperglycemia in the short term but without causing an activation of IGF1R it might be difficult to sustain its beneficial activity. Topical insulin application along with anit-let7b antagomir might be a more potent alternative option that needs to be tried in pre-clinical animal models.

In addition, it seems that there are many different miRNAs that can potentially target *IGF1R*; even with P_CT_>0.75 there are 4 different miRNAs. It will be important to define if different miRNAs are induced under different kinds of burn or are they all induced redundantly and result in down regulation of IGF1R. If multiple miRNAs are induced that target *IGF1R*, it might be an attractive option to identify what causes these upregulation. Identification of that mechanism will provide a better therapeutic handle to regulate and treat hyperglycemia in patients with burn injury.

## MATERIALS AND METHODS

### 30% TBSA animal model

All animal studies were approved by the Institutional Animal Care and Use Committee of The 309th Hospital of PLA. Twelve rats were randomly divided into sham and burn groups (9 rats in burn and 3 rats in sham group, respectively). Post-anesthesia, 30% total body surface area (TBSA) and full-thickness burn model was performed based on previously describe protocols [15, 22]. Briefly, the dorsal hair was shaved before the back skin in the burn groups were placed in hot water (94°C) for 12 seconds. Shock was prevented by simultaneous injection of balanced salt solution at 40 ml/kg body weight. 1% tincture of iodine was administered on the wounds and the area was kept dry. The similar skin region in the sham rats were placed in water at 37°C and similar protocols post-burn injury were performed on the sham group. For six of the 9 rats in the burn group, let-7b antagomir or control antagomir (n=3 for each group) (ThermoFisher Scientific) was injected at 100 fmol/kg body weight through the tail vein post-anesthesia every day for 7 days before induction of burn injury.

### Fasting blood glucose test

Post-fasting for 12 hours (both solid and liquid), blood from tail vein was collected and blood glucose was evaluated using a digital glucometer (ACCU-CHEK Active, Roche, Shanghai) following the manufacturer's instruction.

### Skeletal muscle collection

After modeling for 7 days, anterior tibial muscle and muscle in each group were collected. Blood was obtained from aorta and centrifuged at 1,000 rpm for 15 minutes to collect the serum. Tissues designated for miRNA isolation was stored in RNAlater prior to storage in liquid nitrogen along with specimens for immunofluorescence.

### Cell culture and treatment

L6 rat skeletal muscle cells were obtained from ATCC (Manassas, VA, USA) and maintained in DMEM (4.5g/L glucose, Hyclone, Logan, UT, USA) with 10% FBS, 100U/mL penicillin and 100μg/mL streptomycin at 37°C in 5% CO_2_. To differentiate L6 myoblasts into myotubes, the culture medium was substituted with DMEM plus 2% horse serum after the L6 myoblast cells became confluent. Four days after the initiation of the differentiation, the cells were treated with 10 μM cytosine arabinoside (C1768, Sigma, St. Louis, MO, USA) for 24 hours to remove the dividing myoblasts. Prior to being cultured with serum from burn or sham rats, the myotubes were serum starved for 12 hours.

### Total RNA extraction and quantitative real time PCR (qRT-PCR)

The total RNA from was extracted from the anterior tibial muscle or from serum of burn and control patients with TRIZOL reagents (15596-026, Invitrogen, Carlsbad, CA, USA) according to the manufacturer's instructions, and quantified with ultraviolet spectrophotometry (DU-800, Beckman, Fullerton, CA, USA). The expression levels of indicated miRNAs were done using customized Megaplex™ Primer Pools and TaqMan miRNA arrays (ThermoFisher Scientific). Data was normalized to *U6* snRNA expression and analyzed by the -ΔΔCt method.

### Gene construction

The *IGF1R* 3′ UTR clone in pMirTarget was obtained from Origene. The *IGF1R* 3′ UTR reporter was constructed by amplifying the endogenous *IGF1R* 3′ UTR from the Origene. XhoI and ApaI sites were added to the 5′ and 3′ ends of the fragment during the preceding PCR reaction and cloned into the XhoI and ApaI site on the Rr-luc-6XCXCR4 Renilla luciferase vector (Addgene). To make the *IGF1R* 3′UTR mutant construct, site-directed mutagenesis was used to delete 99-105, 2619-2626, and 6661-6667 regions, corresponding to the predicted let-7b binding sites. A firefly luciferase vector was used as transfection and normalization control in all luciferase assays. Constructs were sequence verified before being used in experiments.

### Transfection and luciferase assays

L6 myotubes (4 × 10^4^), treated with serum from burn rats, were transiently transfected with wild-type and let-7b binding mutant of *IGF1R* luciferase vectors using Lipofectamine LTX (Life Technologies, Beijing, China) as per the manufacturer's instructions. Where indicated, cells were co-transfected with let-7b antagomir. Fourty-eight hours after transfection, the renilla and firefly luciferase activities were measured consecutively using Dual-luciferase reporter assay system (Promega, Madison, WI, USA) as per manufacturer's protocol. Each reporter plasmid was transfected at least twice in triplicate. Post-normalization the data was represented as relative fluorescence units (RFU) ± standard deviation (SD).

### Immunofluorescence analysis

Immunofluorescence was performed using routine protocols as described previously [15].

### Protein extraction and western blotting

Protein was extracted from anterior tibial muscle with RIPA buffer, containing 50 mM Tris-HCl (pH 7.4), 150 mM NaCl, 1% NP-40, 0.25% Na-deoxycholate, 1 mM EDTA, 1% protease inhibitors cocktail (78425, Pierce, Rockford, IL, USA), 1 mM PMSF (36978, Pierce, Rockford, IL, USA), 1 mM Na_3_VO_4_ (S6508, Sigma, St. Louis, MO, USA) and 50 mM NaF (S7920, Sigma, St. Louis, MO, USA). Protein concentration was quantified with a BCA Protein Assay Kit (23227, Pierce, Rockford, IL, USA) according to the manufacturer's instructions. Before the samples were loaded onto the SDS-PAGE gels, 40 μg of total protein was mixed with loading buffer and denatured for 5 minutes at 100°C in a water bath. The protein was transferred to PVDF immuno-blotting membranes using a Electrophoretic Transfer Cell (Mini Trans-Blot®, Bio-Rad, Hercules, CA, USA) after being separated by electrophoresis in a Tetra Cell (Mini-PROTEIN®, Bio-Rad, Hercules, CA, USA). Subsequently, the PVDF blots were incubated in blocking buffer (5% dry fat-free milk in TBST) for 30 minutes followed by incubation for 1.5 hours at room temperature in blocking buffer containing the appropriate dilution of primary antibody (anti-phospho-Akt (Catalogue 4060), anti-Akt (Catalogue 2920), anti-phospho-Gsk3β (Catalogue 5558), anti-Gsk3β (Catalogue 12456), anti-phospho-S6 (Catalogue 4858), anti-S6 (Catalogue 2317), anti-phospho-eIF2B (Catalogue 3596), anti-eIF2B (Catalogue 3595), and anti-GAPDH (Catalogue 5174); Cell Signaling, Danvers, MA, USA). The membranes were washed (4 times at 5 minutes/wash) in TBST and then incubated for 1 hour in blocking buffer containing the appropriate HRP conjugated secondary antibody (ZB-2301, Zhong Shan Golden Bridge Biotechnology, Beijing, China). After washing as described above, the blots were incubated in a chemiluminescent substrate solution (ThermoFisher Scientific, Rockford, IL, USA) for the desired amount of time to visualize the bands.

### Statistical analysis

Results are presented as the mean ± SD. A student's t test was used for statistical analyses. Differences were considered significant at a P value less than 0.05.

## SUPPLEMENTARY MATERIALS FIGURE



## Abbreviations

IGF1R: insulin-like growth factor receptor 1
TBSA: total body surface area
PI3-K: phosphatidylinositol 3-kinase
BAD: bcl-2 antagonist of cell death
FOXO: Forkhead box O
IGF1R: insulin-like growth factor 1 receptor
qRT-PCR: quantitative real time PCR
UTR: untranslated region.
