# Supplementary material for: Differential expression of microRNA let-7b-5p regulates burn-induced hyperglycemia
Source: Oncotarget. 2017 Aug 24;8(42):72886–92. doi: 10.18632/oncotarget.20543 (PMC5641176; doi:10.18632/oncotarget.20543)
Supplement: Supplementary file 1 [file oncotarget-08-72886-s001.pdf]

Differential expression of microRNA let-7b-5p regulates burn-induced hyperglycemia

SUPPLEMENTARY MATERIALS

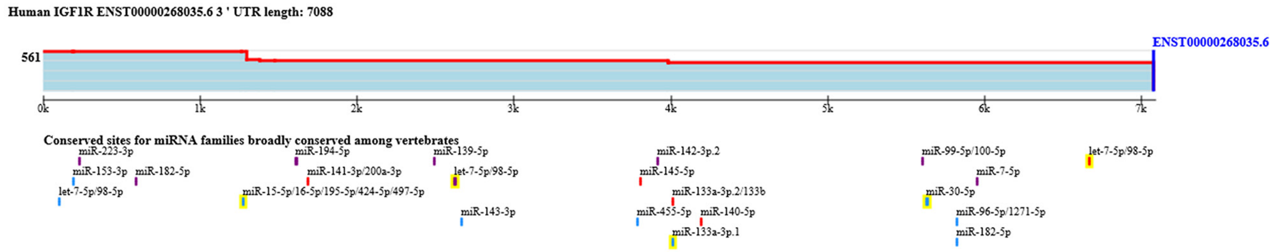

Supplementary Figure 1: Putative miRNA targeting 3'UTR of human *IGF1R* as predicted by TargetScan algorithm. Sites with $P_{CT} > 0.75$ are highlighted with yellow.
